# A Case of Diabetes

**Published:** 1855-08

**Authors:** J. B. Hoag

**Affiliations:** Carthage, Rush Co., Ind.


ARTICLE IV.—*A Case of Diabetes.*

MESSRS. EDITORS:—During my practice in the city of New York, I had the good fortune to cure a bad case of Diabetes, and as this belongs to that class of diseases which have by many been regarded as incurable, I offer a narrative of the facts in the case, and the remedies used, for publication in the columns of your highly interesting and truly valuable Journal, hoping it may be of benefit to some of my professional brethren.

The patient was a young man of about twenty-two years of age. Previous to his being attacked with this disease, he enjoyed the best of health; was robust, and possessed of a large amount of physical strength.

At the time he called on me, he was fearfully emaciated, and in appearance resembled a person in an advanced stage of consumption. He told me that he passed urine some thirty or forty times during every twenty-four hours, to the amount of several quarts, and that it "was as sweet as honey."

After closely questioning him, and informing myself thoroughly relative to his case, I gave him the following prescription, viz:

℞ Sulph. Antimoni Precip. ℈j

Pulo Doveri ℈ij

" Opii qrs. x

" Alumen ℈jv

M et fiat chart no. xx.

Take one at morning and one at night.

I directed him to abstain entirely from eating any kind of fruit, green vegetables, or acid or saccharine substances. On the seventh day after using the above medicine, he called on me, and informed me that he urinated not to exceed five or six times in twenty-four hours, and that his urine was much changed in appearance and taste, and much diminished in quantity, and that his thirst had much abated. I then ordered the following mixture:

℞ Tinct. Canthar, ℥j

" Digitalis ℥j

" Cinchon ℥ij

Mur. Tinct. Ferri. ℥j

Aqua. Pura. ℥iij

M Dose a teaspoonful at noon of each day.

I also directed him to continue the use of the powders. Two weeks after he called again, and informed me that his thirst and appetite were natural; that he felt hearty and strong, and was increasing in flesh. The result was a perfect restoration to his former good health and exercise of his physical powers.

Since then I have treated another case of confirmed Diabetes (though not so far advanced as the first) in the same way, with the same happy result.

Yours with deference and respect, J. B. HOAG, M. D.

Carthage, Rush Co., Ind., June 10, 1855.

